# Angiogenic potency evaluation of cell therapy candidates by a novel application of the in vitro aortic ring assay

**DOI:** 10.1186/s13287-017-0631-1

**Published:** 2017-08-14

**Authors:** Farwah Iqbal, Peter Szaraz, Matthew Librach, Andrée Gauthier-Fisher, Clifford L. Librach

**Affiliations:** 1 The Create Fertility Centre, 790 Bay Street, Suite 412, Toronto, Ontario M5G 1N8 Canada; 2 Department of Obstetrics and Gynecology, University of Toronto, 123 Edward Street, Suite 1200, Toronto, Ontario M5G 1E2 Canada; 3 Department of Physiology, University of Toronto, 1 King’s College Circle, Room 3127, Toronto, Ontario M5S 1A8 Canada; 4 Institute of Medical Sciences, University of Toronto, 1 King’s College Circle, Room 2374, Toronto, Ontario M5S 1A8 Canada

**Keywords:** Angiogenesis, Aortic ring assay, Cell migration, Cellular regenerative therapy, Endothelial networks, Mesenchymal stromal cells, Perivascular cells, Umbilical cord

## Abstract

**Background:**

Due to limitations of current angiogenesis assays, we aimed to develop a novel application of the rat aortic ring assay to assess the angiogenic potential of mesenchymal stromal cells (MSCs). First-trimester human umbilical cord-derived perivascular cells (FTM HUCPVCs) have multipotent characteristics and previously demonstrated angiogenic potential. We compared the effect of this young source of MSCs and adult bone marrow stromal cells (BMSCs) on ex vivo aortic endothelial network formation.

**Methods:**

Thoracic segments of adult rat aortas were isolated, sectioned and embedded into Matrigel™. Fluorophore-labeled FTM HUCPVC lines and BMSCs (*N* = 3) were cocultured with developing endothelial networks (day 0). MSC integration, tube formation and endothelial network growth were monitored daily using phase-contrast and fluorescence microscopy. Quantification of endothelial networks was performed using ImageJ network analysis software on day 5 of coculture.

**Results:**

FTM HUCPVCs from two umbilical cord samples migrated toward and integrated with developing aortic ring tubular networks while displaying elongated morphologies (day 1). In contrast, BMSCs did not show targeted migration and maintained spherical morphologies with limited physical interactions. Within 1 week of coculture, FTM HUCPVC lines contributed to significantly greater radial network growth and network loop formation when compared to BMSCs and untreated networks.

**Conclusions:**

We have developed a novel potency assay to assess the angiogenic potential of cell therapy candidates. Favorable properties of FTM HUCPVCs over BMSCs that we observed with this assay and which merit further study include chemotaxis, affinity for developing vasculature, and physical supportive interactions contributing to the development of endothelial networks.

**Electronic supplementary material:**

The online version of this article (doi:10.1186/s13287-017-0631-1) contains supplementary material, which is available to authorized users.

## Background

Development of new blood vessels is an essential process required for the regeneration of tissue injured by pathological processes including ischemia, inflammation, degeneration and traumatic injury [1, 2]. Development of new therapies to achieve functional tissue regeneration must therefore involve re-establishment and maintenance of healthy blood flow. Several interconnected cellular and molecular mechanisms regulate the three major processes involved in re-establishment of functional circulation for effective tissue regeneration: vasculogenesis, angiogenesis and arteriogenesis [3, 4]. Induction of angiogenesis by delivering therapeutic factors in the form of proteins or genetic materials has been studied extensively and reached clinical trials [5–7]. A significant challenge in this regard has been achieving the efficient delivery of factors such as vascular endothelial growth factor (VEGF), platelet-derived growth factor (PDGF) and/or basal fibroblast growth factor (bFGF) to sites of injury. Consequently, results observed in clinical practice were inconsistent between published studies [8, 9]. This variability may be attributed to the limited longevity of nucleic acid constructs and polypeptides at the target site and finite targets of growth factors, resulting in only transient effects [10, 11]. Thus, for successful regeneration, there is a need for multitargeted approaches with sustained activity. This need to address multiple aspects of vascular formation over a longer period of time explains the shift from growth factor delivery to stem cell-based therapies. Stem cells may have the potential to locally produce angiogenic factors, self-replicate and/or directly differentiate into new blood vessels [12, 13]. Therefore, supplying potential angiogenic supporting cell types with all, or some, of these properties into ischemic tissue holds great promise. The delivery of “adult” stem or progenitor cells has mainly focused on endothelial progenitor cells (EPCs) and mesenchymal stromal cells (MSCs) [14–17].

The lack of a “gold standard” in vitro assay is one of the challenging aspects of studying angiogenesis and evaluating the efficacy of potential new drugs and candidate cell types. An ideal angiogenesis assay must be robust, rapid, reproducible and reliable. It must include assessment of multiple parameters, include positive and negative controls, and most importantly mirror expected preclinical and clinical observations [18]. Although a significant number of in vitro and in vivo assays are available (Table 1) [18–38], they each have various limitations in terms of applicability and feasibility. In general, most in vitro assays evaluate the effects of cells or compounds on endothelial cell migration, proliferation and differentiation into tubular structures, all of which are important for angiogenesis. However, ‘translatable’ assays should also evaluate: efficiency to promote the formation of functional blood vessels; augmentation or replacement of supporting cell types, such as pericytes, smooth muscle cells and fibroblasts, in addition to endothelial cells; and processing of extracellular matrix (ECM) and/or basement membrane. Despite efforts to coculture various vascular cell types together, there has been little success in developing an assay that includes all of the aforementioned. In vivo assays allow the direct implantation of test products into animal models, allowing qualitative and quantitative analysis of angiogenic responses. Limitations of in vivo approaches include animal species restrictions, xeno-immune rejection, complex setup protocols, cost and technical quantification methods [2, 39].

**Table 1:** Available assays to evaluate angiogenic potential

Assay	References	Advantages	Limitations
In vitro			
Endothelial proliferation assays	Gomez and Reich 2003 [19] Yu et al. 2004 [20]	• Reproducible and easy to set up • Provide quantifiable data • Measure proliferation and apoptosis	• Short window of analysis after culture due to endothelial cell senescence • Sensitive to cell density • Involve endothelial cells from macrovascular origin (HUVEC)
Endothelial cell migration assays • Transwell systems • Wound healing assays	Wong and Gotlieb 1984 [21] Schor et al. 2001 [22] Albini et al. 2004 [23]	• Reproducible • Short duration (4–6 hours) • Quantitative analysis of endothelial cell migration over time • Sensitive to small alterations in concentration gradients	• Difficult to define and maintain transmembrane gradients • Inability to observe cell migration in transwell • Challenging to establish matching conditions between control and experimental groups • Difficult to obtain accurate results with low cell counts
Endothelial tube formation assays	Lawley and Kubota 1989 [24] Kanzawa et al. 1993 [25] Auerbach et al. 2003 [26]	• Useful to test angiogenic and anti-angiogenic effects of compounds • More representative of in vivo angiogenesis than 2D assays • Introduce ECM to culture system • Measure proliferation and differentiation	• Lack of consistent lumen formation • Homogeneous tubules • Sensitive to uneven matrix coating of wells and cell density • Time-consuming analysis (multiple parameters of analysis)
Ex vivo			
Chick aortic arch model	Staton et al. 2009 [18]	• System includes nonendothelial cells (pericytes, smooth muscle cells) and ECM • Easy set up, early cell outgrowth (48 hours) • Embryonic endothelial cells resemble microvascular phenotype	• Endothelial cells are in a proliferative state in the embryo (not representative of true in vivo scenarios)
In vivo			
Chick chorioallantoic membrane assay	Ribbati et al. 1995 [27] Ejaz et al. 2004 [28]	• Simple and inexpensive • Ideal to implant tissue or organ grafts • CAM membrane is immunoprivileged enabling xeno-graft studies • Ideal for large-scale screening • Noninvasive observation	• CAM has endogenous vasculature, difficult to distinguish pre-existing and newly formed vasculature • 11-day incubation time prior to implantation of test reagents • Incision in shell may induce inflammation and a specific angiogenic response • Sensitive to oxygen tension
Matrigel™ plug assay	Passaniti et al. 1992 [29] Baker et al. 2006 [30]	• Quantitative histological analysis • Matrigel™ provides natural environment for angiogenesis	• Time consuming, including 2 weeks of plug incubation in host, isolation, sectioning, analysis • Costly
Sponge/matrix implant assay	Salvatore et al. 1961 [31] Dellian et al. 1996 [32]	• Include defined polymers to study angiogenesis • Angiogenic response can be monitored over time in live animals • Ideal for studying tumor-induced angiogenesis	• Implants can become encapsulated with cytokine-secreting macrophages • Inflammatory response may interfere with angiogenic response • Undesirable fibrosis • Variable retention of test compounds in different substrates
Corneal assay	Gimbrone et al. 1974 [33] Muthukkaruppan and Auerbach 1979 [34]	• New blood vessels easily observed due to the absence of background blood vessels • Executed in mice, rats, rabbits • Noninvasive monitoring and data easily quantifiable	• Challenging surgical procedure • Limited space of test substance injection • Inflammation difficult to avoid • Atypical angiogenesis because cornea is avascular • Costly
Dorsal air sac model	Selye 1953 [35] Oikawa et al. 1997 [36]	• Adaptable to various applications • Allow continuous non invasive monitoring of endothelial networks	• Difficult to distinguish pre-existing and newly formed vasculature • Delicate procedure (irritation to dorsal skin)
Zebrafish assay	Rubinstein 2003 [37] Isogai et al. 2001 [38]	• Enables large-scale projects • Shared angiogenic genes and mechanisms • Easily monitored/quantified • Ideal for testing of anti-angiogenic compounds • Suitable for genetic studies of angiogenesis	• Relevance of fish endothelial cell angiogenesis is under debate • Nonmammalian cell types and involves embryonic cells • Costly to maintain breeding conditions

*CAM* Chick chorioallantoic membrane assay, *ECM* extracellular matrix, *HUVEC* Human umbilical vein endothelial cells

**Fig. 1:**
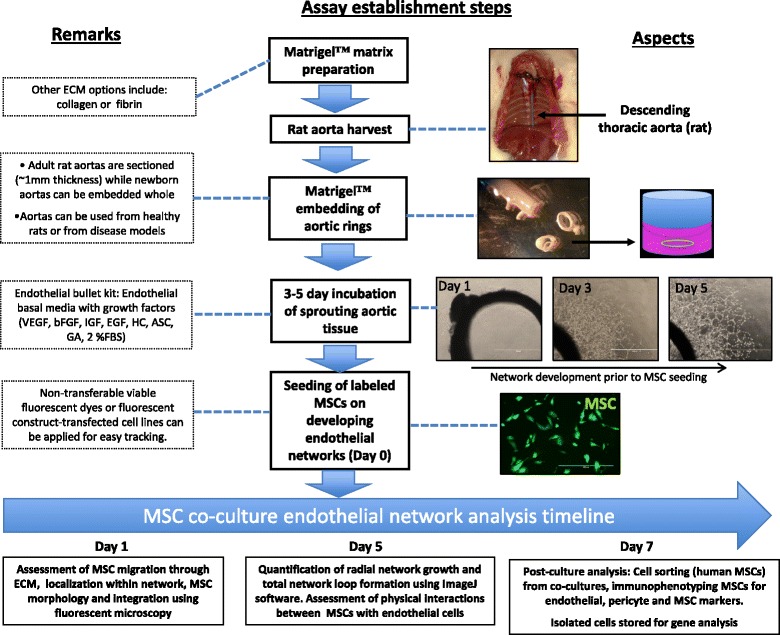
General protocol to set up novel application of the aortic ring assay. Main steps for set up and analysis of MSC cocultures with the aortic ring assay (*solid boxes*) and additional notes (*dotted boxes*)*. bFGF* basal fibroblast growth factor, *ECM* extracellular matrix, *FBS* fetal bovine serum, *FGF* fibroblast growth factor, *MSC* mesenchymal stromal cell, *VEGF* vascular endothelial growth factor, *IGF* insulin-like growth factor, *HC* hydrocortisone, *ASC* ascorbic acid, *GA* gentamicin, amphotericin B

Due to the shortage of available angiogenesis assays, we propose a modified application of the previously described aortic ring assay [40]. The aortic ring assay was first reported in 1990 by Nicosia and Ottinetti [41] as a unique ex vivo angiogenesis assay, having clear advantages over other in vitro assays. Advantages of this assay include: easy to observe tubular structures; accessory supportive cells (smooth muscle cells, fibroblasts and pericytes); ECM from host and/or supplied (fibrin); endothelial cells not preselected by passaging and therefore are in a nonproliferative state; lack of inflammatory components; and quick and inexpensive set up [42–44]. Typically, the aortic ring assay is used to test the angiogenic potential of small secretory proteins [45, 46] and pharmacological agents [47, 48], and evaluate angiogenic responses of transgenic mouse models following genetic alteration of key angiogenic factors [49, 50]. Earlier research articles focused on the contribution of aortic tissue resident nonendothelial cell types to the angiogenic response, such as resident macrophages and mural smooth muscle cells, or evaluated the reaction of tumor aggregates with the aortic ring-derived endothelial networks [43]. We present a novel approach to study the angiogenic effect of potential candidates for regenerative cell therapy (Fig. 1). Compared to the article by Nicosia and Ottinetti [41], we present a method to study homing, integration and network developing properties of therapeutic candidate cell types, with the addition of performing downstream analysis including immunophenotyping and gene expression profiling of both endothelial cells and administered human cells (Table 2).

**Table 2:** Comparison of aortic ring assay applications and novelty

	Nicosia and Ottinetti 1990 [41]	Present study 2017
Application/novelty	• Developed a quantitative and reproducible angiogenesis assay for cell-free compounds • Tested the effect of inhibitory/stimulatory soluble factors on angiogenesis	• Modified a quantitative and reproducible angiogenesis assay for coculturing cells • A novel application to study the angiogenic function and potency of cell therapy candidates for clinical application and banking
Set up	• Thoracic rat aorta embedded in fibrin or collagen • Prepared agarose wells to support aortic rings • Endothelial media: MCDB 131 to a mixture of 1:1 with DMEM F12 (FBS-free) • Stimulation of angiogenesis with mouse sarcoma 180 • Studied inhibition of angiogenesis using hydrocortisone	• Thoracic rat aorta embedded between two layers of Matrigel™ • Support of angiogenesis from EHS mouse sarcoma extract (Matrigel™) • Endothelial basal media supplemented with 2% FBS and 1% penicillin/streptomycin for cocultures • Prelabeled MSCs cocultured with developing endothelial networks for tracing
Analysis	• Daily counting of newly developed microvessels (15 days) using bright-field microscopy • Florescent imaging of endothelial networks using FVIII-Ra immunohistochemistry	• Quantified radial network growth and network loops at day 5 following MSC coculture • Live-cell florescence imaging of MSC migration, integration site preference and morphology (coverage) in endothelial networks • Developed methods to extract MSCs and endothelial cells to perform downstream analysis including flow cytometry and qPCR

*FBS* fetal bovine serum, *MSC* mesenchymal stromal cell

MSCs have received significant attention in the field of cell-based regenerative medicine and cancer treatment due to their multifaceted regenerative properties, including the modulation of angiogenic processes [51–54]. While MSCs can be isolated from virtually any vascularized tissue in the body, bone marrow-derived mesenchymal stromal cells (BMSCs) are the most studied candidate for both autologous and allogeneic cell therapy [55]. BMSCs regulate hematopoietic stem cell (HSC) proliferation and differentiation, contribute to blood vessel formation and improve tissue function, particularly in the cardiac muscle [56–59]. Despite clear advantages of autologous stem cell therapy, BMSC therapy is limited by cell senescence-mediated reduction in differentiation potential and time constraints in collection and propagation protocols [60, 61]. Importantly, many studies have demonstrated an age-associated decline in the number and function of host-derived stem cells, limiting the effectiveness of autologous stem cell therapy in aged patients [62, 63]. The use of nonautologous cells from younger sources for transplantation, especially in older recipients, may overcome these challenges. Our group is currently investigating human umbilical cord perivascular cells (HUCPVCs) derived from the perivascular region of the human umbilical cord (HUC). These cells represent an accessible and rich source of young MSC populations with pericyte-like properties, and have been characterized from both first-trimester (FTM) and term umbilical cords [64–67]. FTM HUCPVCs have increased expansion potential, as well as immunoprivileged and multipotent properties [66], and preliminary experiments suggest that HUCPVCs promote significant cardiac regeneration and improve cardiac function following myocardial infarction when compared to BMSCs [68].

Here we present a novel application of the aortic ring assay to assess the ability and potency of cellular therapy candidates to mediate ECM processing, migrate to areas of angiogenesis and contribute to vessel development through physical contact. As model cell types, we aimed to compare ontogenetically early (prenatal) and late (adult) sources of human MSCs, human FTM HUCPVCs and human BMSCs in the aortic ring assay.

## Methods

### Use of animals

All animal procedures were conducted and reported according to ARRIVE guidelines and approved by the Animal Care Committee of the University Health Network (Toronto, Canada). All studies were performed with institutional research ethics board approval (AUP 3220.5, University of Toronto, Toronto, Canada). Aortic tissues were isolated from Sprague–Dawley female rats of reproductive age. Animals were euthanized in carbon dioxide chambers set to 20% gas replacement (flow rate = chamber volume × 0.2 per minute). The aorta was exposed by an excision through the chest cavity and removal of lung tissue. The aorta was identifiable adjacent to the vertebral column and white in color. Using surgical tools, the thoracic aorta was excised and sectioned into ~1 mm sections yielding approximately 15–20 rings. To account for variability between animals, each experiment was repeated three times (*N* = 3).

### Use of matrix

Matrigel™ (Corning) was selected for the assay due to the basement membrane-like composition [69]. Matrigel™ (200 μl) was coated evenly on 12-well plates (on ice) and then placed in a humidified incubator (37 °C, 5% CO_2_) for 30 minutes. Once Matrigel™ was polymerized; a freshly obtained aortic ring was placed at the center of each well. Then 300 μl of Matrigel™ was carefully applied on top of the aortic ring tissue and incubated for 30 minutes (37 °C, 5% CO_2_). Once polymerized, 1000 μl of prewarmed endothelial growth media (EGM) (see next section) was added to each well and then placed back into the incubator and intermittently observed until endogenous endothelial networks developed (3–5 days).

### Use of media

Following the final Matrigel™ polymerization, the aortic ring sandwich assay was incubated in 1000 μl of Endothelial Growth Media-2 (EGM-2™) BulletKit™ (Lonza catalog no. CC-3162). EGM-2 was removed 24 hours following incubation and replaced with 1000 μl of endothelial basal media (EBM) supplemented with 2% fetal bovine serum (FBS) (Hyclone) and 1% penicillin/streptomycin (P/S) (Gibco) for the remainder of the assay and replaced every 2 days.

### Cell culture of FTM HUCPVCs and BMSCs

FTM HUCPVCs (8–9 weeks of gestation) were isolated as described previously [66]. FTM HUCPVCs were expanded in StemPro® MSC SFB XenoFree culture media supplemented with 1% StemPro® SFM XenoFree Supplement, 1% l-glutamine and 1% P/S. Prior to culture, 10-cm^2^ culture dishes (Corning) were coated with 7 ml of PBS containing Ca^2+^, Mg^2+^ and 1% of CELLstart™ humanized substrate for stem cell culture (Thermo Fisher Scientific) and placed in the incubator for 1 hour at 37 °C, 5% CO_2_. For the assay, passage 3 (P3) of two FTM HUCPVC lines (FTM 1 and FTM 2, two donors) were thawed and plated at a density of 2.5 × 10^3^ cells/cm^2^ in a 10-cm^2^ culture dish and incubated at 37 °C, 5% CO_2_. FTM HUCPVCs were passaged at 70–80% confluency and cocultured with aortic ring assay at P4. The BMSC line were purchased commercially (Lonza) and expanded in Minimum Essential Medium Eagle with alpha modifications (α-MEM), 10% FBS and 1% P/S. BMSCs were cultured at a density of 5.0 × 10^3^ cells/cm^2^ in 10-cm^2^ culture dishes. BMSCs were passaged at 70–80% confluency (P4) and cocultured with the aortic ring assay at P4 (one donor).

### Coculture set up

For coculture, MSCs were harvested and prepared as single-cell suspensions using TrypLE (Invitrogen) at 37 °C for 5 minutes. MSCs were prestained with cell tracker green dye (CTG) (CellTrackerGreen™; LifeTechnologies) for 30 minutes, washed and resuspended in EBM + 2% FBS, 1% P/S. Using an automated cell counter (Invitrogen), approximately 10,000 prestained MSCs were cocultured with aortic ring assay endothelial networks (day 0).

### Imaging and quantification of endothelial networks

**Fig. 2:**
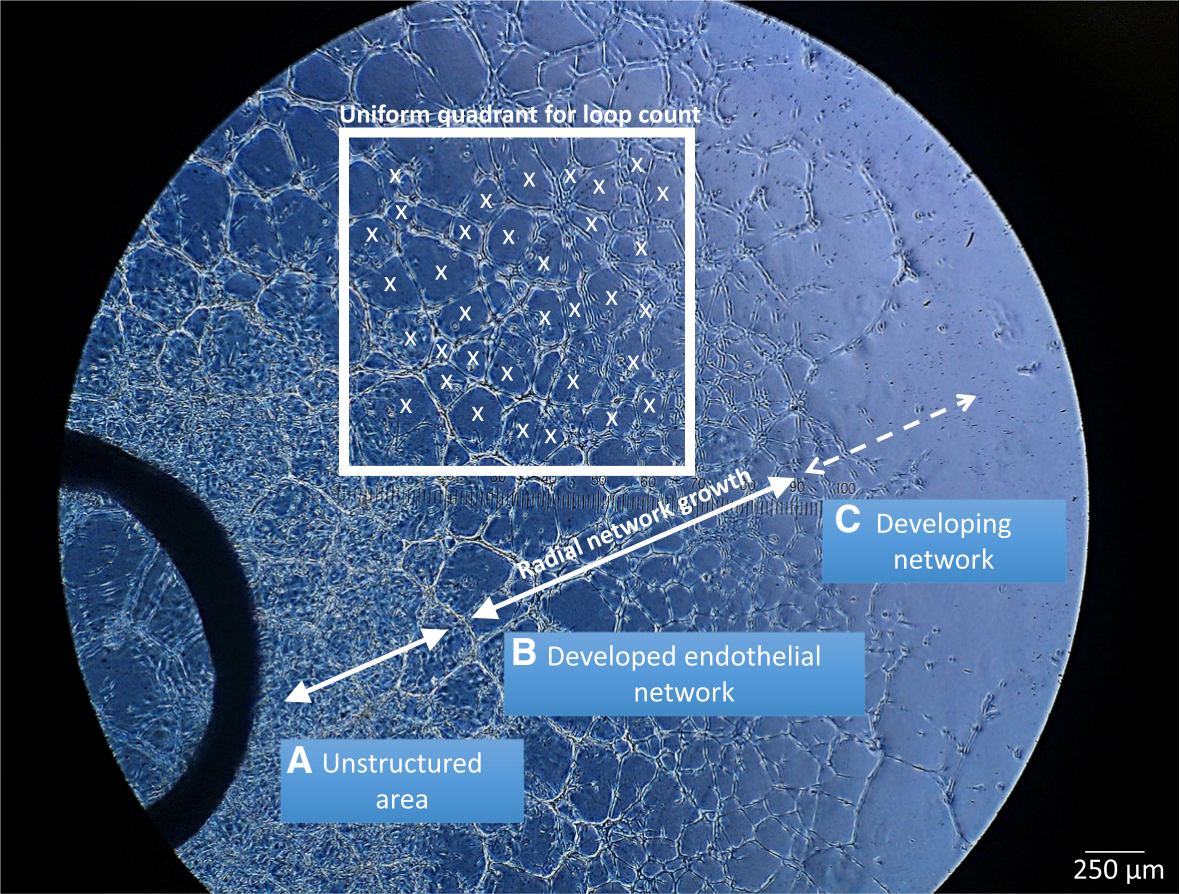
Representative image of aortic ring network analysis. Endothelial networks are divided into three concentric regions based on structure: unstructured area in close proximity to aortic ring tissue (**a**), developed/structured endothelial networks (**b**) and developing networks located in the periphery of the ex vivo tissue culture (**c**). Radial network growth and uniform quadrant for loop count are defined within the developed endothelial network (**b**). *X* closed endothelial loop counted in uniform quadrant. *Scale bar* = 250 μm

Following 24 hours of incubation, bright-field (Olympus) and fluorescence microscopy images (EVOS™; LifeTechnologies) of aortic ring cocultures were taken to evaluate MSC-mediated ECM processing and migratory potential. The preferential homing of MSCs to unstructured proliferating areas (Fig. 2a), previously developed endothelial networks (Fig. 2b) or newly developing endothelial networks was assessed (Fig. 2c). These imaging methods also documented overall network development, and physical interactions between MSCs and endothelial cells. Phase-contrast images of four fields were taken to measure radial network growth and the total number of network closed loops up to 7 days (Fig. 2). The quantification of network growth and network loop formation was quantified using program ImageJ, utilizing the angiogenesis plugin on day 5 of coculture [70].

### Statistical analyses

All results were generated from three independent experiments (*N* = 3) with two rings for each treatment group (*n* = 2). Data were analyzed using Prism™ software (GraphPad). Results were presented as mean ± standard deviation (SD), unless otherwise indicated. Statistical significance was determined using a one-way ANOVA test. Post-hoc analysis was calculated with Tukey’s test for pairwise comparison. Differences in overall network growth and loop formation following coculture were considered significant when *p* ≤ 0.05.

## Results

### Fluorescence microscopy allows qualitative measurements of MSC migration, integration and morphology in conjunction with developing endothelial networks in the aortic ring assay

**Fig. 3:**
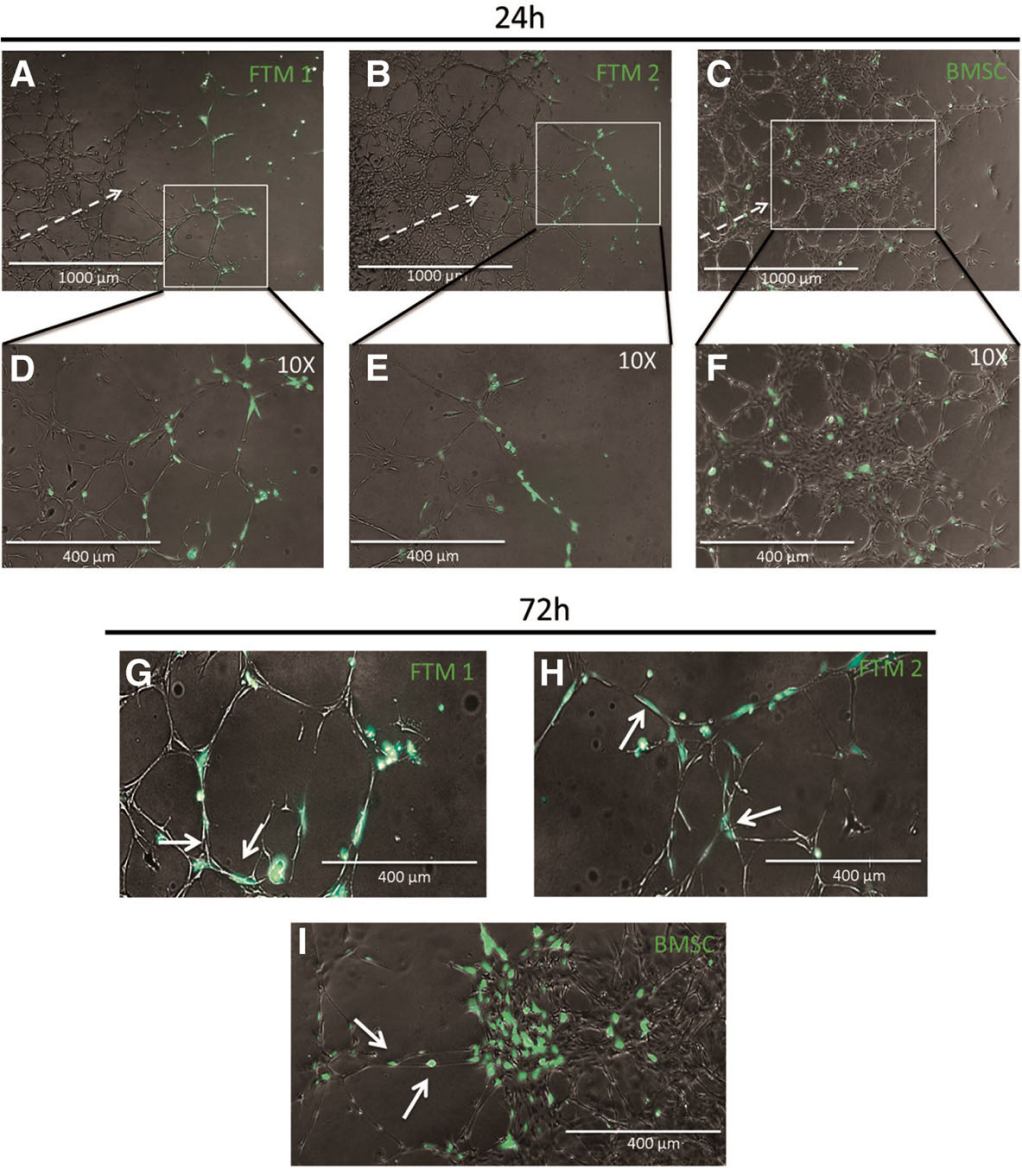
Fluorescent imaging of network region-dependent integration of human MSCs in the aortic ring assay after 24 and 72 hours. Prestained (CellTrackerGreen™) FTM HUCPVCs and FBS containing media-expanded BMSCs added to developing aortic ring endothelial tube networks. Fluorescence microscopy images taken 24 hours after establishing MSC cocultures. FTM 1 and FTM 2 migrate through ECM and home to peripheral developing endothelial networks (**a**, **b**). Higher magnification images display elongated morphologies of FTM HUCPVCs while in close contact with endothelial networks (**d**, **e**). Fewer BMSCs process ECM and home to endothelial networks with no observable preference to peripheral developing networks (**c**). BMSCs display spherical cell morphologies (**f**). High-magnification fluorescence microscopy images of prestained MSCs in rat aortic ring assay following 72 hours of coculture (**g**, **h**, **i**). FTM 1 and FTM 2 display elongated morphologies while displaying endothelial coverage through direct cell-to-cell interactions with endothelial cells (*solid white arrows*) both in network nodes and tubules (**g**, **h**). BMSCs maintain spherical cell morphologies clustered in endothelial network nodes (**i**). *Broken arrow* shows direction of endothelial network growth from aortic ring tissue. Low-magnification images, *scale bar* = 1000 μm; high-magnification images, *scale bar* = 400 μm. *BMSC* bone marrow stromal cell, *FTM* first trimester (Color figure online)

Human MSCs were prestained and cocultured with ex vivo cultured rat aortic rings in order to observe MSC migration in Matrigel™ and homing preferences to endothelial networks (Fig. 3a–f). CTG-positive cells were imaged 24 hours following cell administration. Both FTM 1 and FTM 2 localized at the periphery of developing endothelial networks while displaying elongated cell morphologies. FTM HUCPVCs appeared to migrate to newly developing networks and contributed to the further development of endothelial networks (Fig. 3a, b, d, e). Alternatively, BMSCs demonstrated minimal site preference and homed to areas with limited endothelial tube formation and sites with significant cellular proliferation (Fig. 3c). High-magnification images of BMSCs in coculture displayed spherical morphologies when compared to FTM HUCPVC cocultures (Fig. 3f).

Following 72 hours of MSC administration, high-magnification fluorescent images were taken to further observe cell-to-cell interactions between MSCs and endothelial cells, as well as cellular morphologies (Fig. 3g–i). Both FTM HUCPVC lines (FTM 1 and FTM 2) demonstrated extensive cell-to-cell interactions with endothelial cells (Fig. 3g, h, white arrows) while displaying elongated morphologies typically observed in pericyte–endothelial interactions. Conversely, BMSCs were localized mainly in endothelial proliferation sites and seldom found localized within endothelial tubes or loops (Fig. 3i, white arrows). BMSCs displayed spherical morphologies, suggestive of a physically less supportive “pericyte” phenotype.

**Fig. 4:**
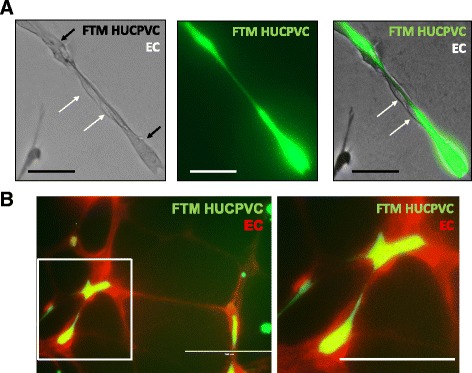
High-magnification fluorescence microscopy images of pericyte–endothelial-like physical interactions. Unstained endothelial cells (*white arrows*) in a single line associated with continuous protrusions connecting prestained FTM HUCPVCs (*black arrows*) (**a**). Fluorescent images of prestained endothelial networks (*red*; CellTrackerOrange™) with prestained FTM HUCPVCs (*green*) demonstrating endothelial and pericyte-like interactions (**b**). Low-magnification images, *scale bar* = 200 μm; high-magnification images, *scale bar* = 100 μm (Color figure online). *EC* endothelial cell, *FTM HUCPVC* first-trimester human umbilical cord perivascular cell

High-magnification images depict physical interactions between endothelial cells and FTM HUCPVCs in endothelial networks with prestained FTM HUCPVCs (Fig. 4a, green) adhering to unstained endothelial cells (Fig. 4a, white arrows). Alternatively, both FTM HUCPVCs (green) and endothelial cells (red) were loaded with fluorescent fluorophore prior to coculturing (Fig. 4b). Both settings revealed elongated connections between the two cell types in the network, suggesting cooperation and supportive cell behavior.

### Bright-field microscopy allows quantitative measurements of overall endothelial network growth when cocultured with MSC treatment groups

**Fig. 5:**
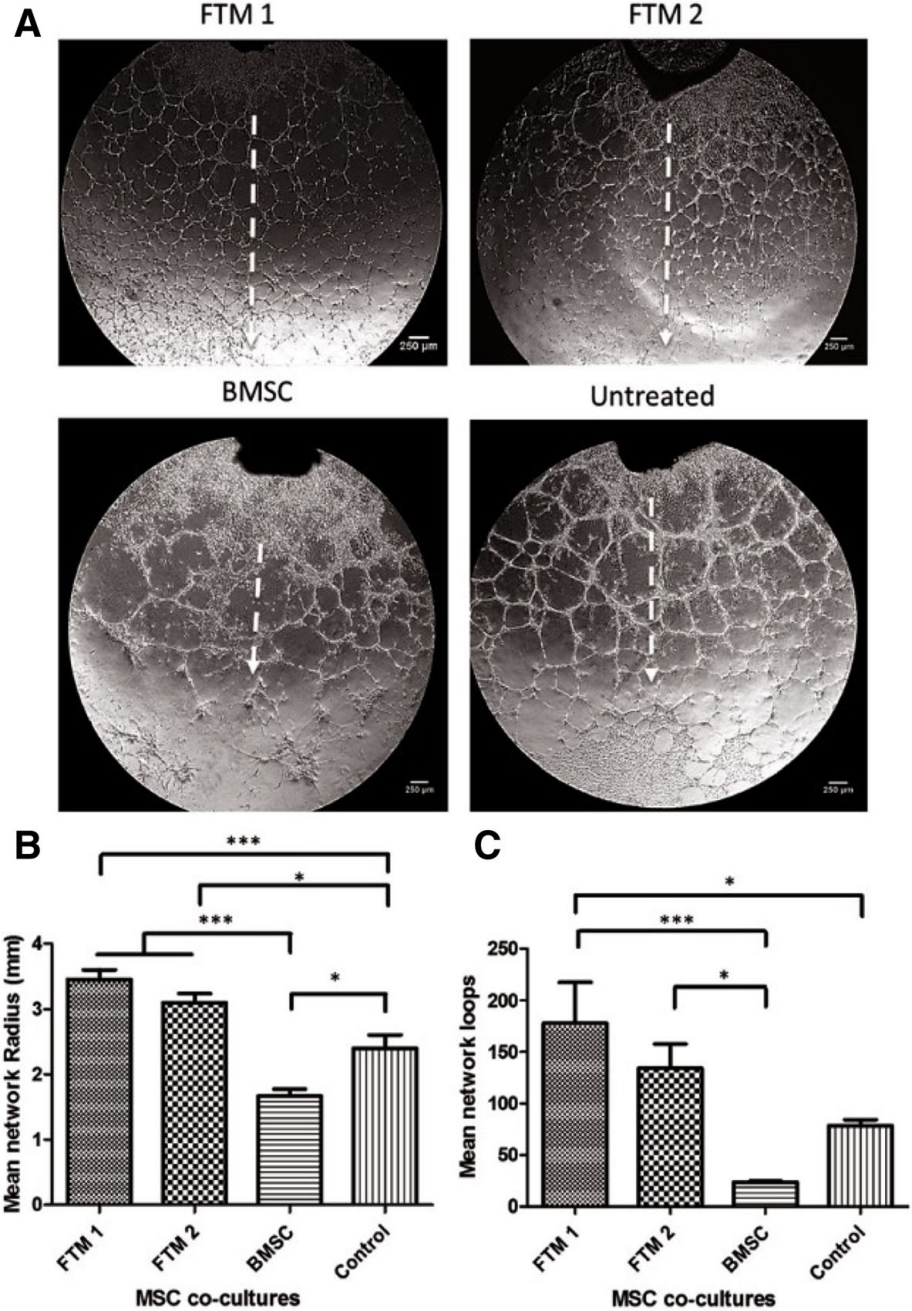
Quantification of endothelial networks at day 5 of MSC coculture with the aortic ring assay. Phase-contrast microscope images of MSC treatment groups taken at day 5 following MSC coculture and utilized to quantify network properties including mean network growth and mean network loop formation (**a**). Both FTM HUCPVCs contributed to greater network growth when compared to BMSC cocultures (*p* ≤ 0.001). FTM 1 and FTM 2 contributed to greater network growth when compared to untreated networks (*p* ≤ 0.001, *p* ≤ 0.05 respectively). BMSC cocultures contributed to inferior network growth when compared to untreated endothelial networks (*p* ≤ 0.05) (**b**). *p* calculated using one-way ANOVA as *p =* 0.0001 using Tukey’s post test (*N* = 3, *n* = 2). Network loops with at least four closed sides were quantified (**c**). Both FTM 1 and FTM 1 cocultures developed greater network loops when compared to BMSC cocultures (*p* ≤ 0.001, *p* ≤ 0.05 respectively). There was no statistical difference between BMSCs and untreated aortic networks. FTM 1 contributed to greater closed loops when compared to untreated networks (*p* ≤ 0.05). Average of four fields of endothelial networks quantified. *p* calculated using one-way ANOVA as *p* = 0.0008 using Tukey’s post test (*N* = 3, *n* = 2). *Scale bar* = 250 μm. For pairwise comparison: *p ≤ 0.05,***p ≤ 0.001. *BMSC* bone marrow stromal cell, *FTM* first trimester

Quantification of endothelial network properties was performed at day 5 of MSC cocultures. Mean network growth was calculated as the distance from proximal closed network loops closest to the aortic ring to the furthest distal closed loops (radius size) (Fig. 5a). Radial growth of endothelial tubular networks within the ECM (Matrigel™) can translate into the penetration range of newly developing vasculature in a regenerating tissue. Images were acquired and collected in a blinded manner and three radius measurements were performed per image. The mean network growth of MSC–endothelial cocultures was as follows: FTM 1, 3.45 ± 0.4 mm; FTM 2, 3.1 ± 0.3 mm; and BMSCs, 1.7 ± 0.3 mm. Untreated endothelial networks developed a mean radius of 2.4 ± 0.5 mm. Statistical comparison between MSC treatment groups demonstrated that both FTM 1 and FTM 2 significantly increased network growth compared to BMSCs (*p* ≤ 0.001) and untreated networks (*p* ≤ 0.001 and *p* ≤ 0.05 respectively). Untreated endothelial networks developed significantly more than BMSCs containing cocultures (*p* ≤ 0.05). There was no significant difference in radial network growth between both FTM HUCPVC lines (Fig. 5b).

In addition to the assessment of the average growth of endothelial networks, endothelial network structure was also analyzed. Analysis of the formation of closed endothelial loops is of great importance because capillary networks consisting of more loops are able to supply a greater surface area, potentially leading to optimal tissue perfusion in vivo [71]. Uniform quadrants were defined for measurement of the complete aortic ring endothelial network. The total numbers of loops were calculated in each quadrant and averaged into one measurement of total loop formation (Fig. 5c). The average number of closed loops for FTM HUCPVC treated cocultures was 178 ± 96 and 134 ± 58 for FTM 1 and FTM 2 respectively, 23 ± 2 for BMSCs and 79 ± 14 for untreated rings. FTM 1 and FTM 2 HUCPVC treated networks contributed to greater endothelial loop formation when compared to BMSCs (*p* ≤ 0.001 and *p* ≤ 0.05 respectively). The average number of closed loops of FTM-1-treated aortic ring networks was significantly greater than untreated networks (*p* ≤ 0.05).

## Discussion

Cell-based therapy is currently the most extensively studied approach for therapeutic angiogenesis. Due to conflicting results reported by clinical studies, there is a lack of consensus regarding the ideal candidate cell type(s) for therapeutic neovascularization. An ideal cell candidate for vascular regeneration should home to the injury site, subsist in the regenerating environment and contribute to ECM remodeling to promote the development of microvasculature [16, 72, 73]. The exact mechanisms of how different stem or progenitor cells contribute to vessel formation are under investigation. Candidate cell types may either indirectly contribute to vascular regeneration by secreting paracrine factors, recruit host progenitor and supportive cell types, and provide physical support to developing vasculature, and/or directly differentiate into endothelial-like cells [74]. A major challenge preceding in vivo studies of angiogenesis is selection of appropriate functional assays that can accurately and efficiently evaluate the therapeutic agent in vitro. Due to limitations of available angiogenesis assays (Table 1), typically more than one assay is used when testing potential angiogenic therapies. An ideal functional assay to evaluate regenerative cell types should be robust, rapid, reproducible, quantitative and reflect the physical and cellular components that determine angiogenesis in vivo [75].

Here, we proposed a novel application of the aortic ring assay that can be utilized to study potential mechanisms involved in angiogenesis and assess the potency of cell candidates for regenerative therapy. For these studies, we compared the angiogenic potential of two independent FTM HUCPVC lines with commercially available adult BMSCs in vitro. We developed a novel method that involves coculturing MSCs with rat aortic endothelial networks to quantify net effects on endothelial network development. Following 24 hours of coculture, fluorescence microscopy demonstrated that FTM HUCPVCs home and integrate into peripheral sprouting endothelial networks and display significant endothelial coverage. BMSCs did not display preferential homing and displayed minimal cell-to-cell interactions with endothelial cells. Both FTM HUCPVC line cocultures contributed to greater network growth when compared to BMSC cocultures and untreated networks (*p* ≤ 0.05), and both FTM HUPCVCs lines contributed to significantly greater network loops when compared to BMSCs (*p* ≤ 0.05). Our test also suggested a difference between FTM HUCPVC lines: cocultures using FTM 1 developed significantly greater loops when compared to untreated networks. Our experiments suggest that ontogenetically young FTM HUCPVCs may have greater angiogenic potential when compared to older BMSCs and that variability between cell lines of the same tissue origin is also detectable with this method.

Angiogenesis is a multistep process typically initiated by injury or tissue remodeling, where endothelial cells degrade the basement membrane using proteases, migrate and proliferate into extracellular space [76]. Once new vessels are initiated, pericytes and smooth muscle cells stabilize and support immature vessels. These interactions between endothelial cells and mural cells are crucial for the transition of immature vasculature into mature and functional vasculature [77, 78]. FTM HUCPVCs are a young source of MSCs with pericyte-like properties. Supporting endothelial cells and vasculature is the inherent function of HUCPVCs in the umbilical cord. FTM HUCPVCs express pericyte-associated markers including CD146, NG2 and PDGFR-β [66]. These characteristics likely contributed to the positive effect we observed on endothelial network development in vitro as well as the intercellular interactions and supportive morphology of FTM HUCPVCs. CD49f is known to be a regulator of both cell-to-cell membrane adhesion [79, 80] and, via its laminin binding potential, cell-to-basal membrane adhesion [81]. We have shown previously that HUCPVCs have an increased expression of CD49f [82], with FTM HUCPVCs expressing the highest level of CD49f. Although BMSCs express vascular cell adhesion molecule-1 (VCAM-1), CD146, PDGF-β and smooth muscle α-actin [83–85], we observed minimal supportive elongated morphology, possibly explaining the significant decrease in radial network growth and network loop formation when compared to FTM HUCPVCs. Interestingly, BMSC cocultures displayed a significant reduction in network radial growth (*p* ≤ 0.05) and endothelial loop formation when compared to untreated networks. It has been shown that the regression of developing microvascular networks depends less on endothelial cell death than endothelial cell arrangement and disarrangement [86]. This can explain our observation that a cell type not contributing to the structured growth of the network causes network regression. BMSCs did not integrate with structured or developing endothelial networks but localized with unstructured endothelial cell masses, causing decreased network development compared to untreated aortic rings. In addition to insufficient physical interactions between BMSCs and endothelial cells, BMSCs may not have provided the optimal growth factors for network development or may even sequester them from cocultures.

Endothelial cells embedded within ECM proliferate and arrange into tubular networks. This is due to availability and diffusion of oxygen and nutrients and also the establishment of microenvironmental gradients [87]. We observed extensive cell proliferation in the vicinity of the aortic tissue, resulting in large cell clusters (Fig. 2a). BMSCs may secrete factors that induce endothelial cell proliferation, thus interfering with endothelial network development. A potential regenerative cell type that indirectly contributes to vascularization should demonstrate beneficial paracrine activity that can fulfill the growth factor demand as well as provide physical support to endothelial cells to sustain endothelial network development. Our results demonstrate the supportive nature of FTM HUCPVCs, contributing to overall network development and stability. We are currently investigating changes in gene expression of critical angiogenic factors following coculture with endothelial cells and also whether the supportive properties of FTM HUCPVCs translate into the promotion of mature and functional vasculature in animal injury models.

One of the key advantages of using the aortic ring assay is the ability to address multiple research questions. To critically analyze the importance of paracrine properties of MSCs in addition to physical cell-to-cell interactions, this assay can also be set up in a transwell system. Thus, the developing endothelial networks and MSCs can exchange secreted paracrine factors while being separated physically. Assay media from cocultures can be analyzed for candidate angiogenic factors using commercially available proteome profilers. Further analysis to determine the fate of multipotent candidate cell types could also be conducted by disrupting cocultures at the endpoint of the assay to sort out human cells from cocultures. Costaining cells with a human cell surface marker (TRA-1-85) and markers of interests can indicate whether candidate cell types have altered expression of multipotency and mesenchymal markers such as CD146 and/or upregulated expression of endothelial markers like CD31 (Additional file 1: Figure S1).

Besides its multiple advantages, there are notable limitations of the described assay. Users need to account for the lag period between embedding of the ex vivo aortic tissue and the initial network development that marks the time of cell administration. Quantification of endothelial network properties can be time consuming but can be resolved by assigning hallmark parameters, including radial growth, tube length and network mesh dimensions. Network analysis applications (such as ImageJ) can significantly decrease the time required for consistent and reliable quantification. Variability between each assay can occur as a result of slight inconsistencies in animal tissue source and handling by the operator. We found that this challenge can be efficiently overcome and quantification becomes statistically reliable by setting up triplicates for each treatment group. Lastly, it is difficult to extract and sort cells of human and rat origin from the embedding Matrigel™ substrate for post-assay analysis. However, specific materials including the Matrigel™ specific enzyme dispase and cell recovery solution can be applied to recover cells for immunophenotypical or genetic analysis (Table 3).

**Table 3:** Aortic ring assay

Advantages	Limitations
• Cost-effective because the aorta is waste tissue from endpoint animal studies • From one aorta, a high yield of replicates can be obtained (approximately 20 rings per adult animal) • Rapid set up including aorta isolation and embedding • Evaluates key steps of angiogenesis including matrix degradation, cell migration, proliferation and morphogenesis into tubular endothelial network • In addition to endothelial cells, includes cell types important for angiogenesis such as resident pericytes and fibroblasts • Endothelial cells have not been preselected by passaging and thus are in a quiescent state at starting point of the assay, reflecting in vivo conditions • Quiescent endothelial cells respond by proliferating and differentiating into tubular networks • Evaluates properties of cocultured candidate cell types including the ability to respond to signals of aortic tissue, induce migration, ECM processing and homing to endothelial networks • Cell-to-cell connections can be observed using fluorescence microscopy • Net effects on angiogenesis can be quantified using image analysis software to assess various network properties (network radial growth, loops, branches and nodes)	• Vessel outgrowths occur from a major vessel while in vivo angiogenesis occurs typically from micro vessels • Takes 3–5 days for initial endothelial network to develop • Variability in angiogenic response can also occur between animal’s due to strain, age and gender • Lack of blood flow (limitation shared with other in vitro and ex vivo angiogenesis assays) • Angiogenic vessel growth is in three dimensions, rendering imaging and quantification difficult • Outgrowth vessels regress over time (2 weeks), thereby limiting long-term analysis

*ECM* extracellular matrix

Cell products prepared for in vivo applications are tested routinely for identity and purity prior to long-term storage. However, high viability and purity may not always translate into greatest functionality [88, 89]. Storing allogeneic cell lines without prescreening for functionality is problematic. Therefore, robust potency assays are required. One of the limitations for cell therapy is donor-based variability. In our laboratory, FTM HUCPVCs from umbilical cords are isolated at 8–10 weeks of gestation. Because interpatient variability can impact the regenerative potential of cell lines, established cell lines are selected for in vivo applications based on their expansion properties (culturing efficacy) and immunophenotyping results. We also employ various functional in vitro assays, including a transbasal membrane invasion and lymphocyte activation assays, to determine optimal cell lines as well as optimal culture conditions for the expansion of FTM HUCPVCs. In order to assess potential functional differences between cell lines, we tested two different FTM HUCPVCs lines and compared their angiogenic potential to BMSCs, a cell type currently under investigation in numerous clinical trials [90], using this modified aortic ring assay. Overall, this new application revealed greater differences in angiogenic potential between cell types from different origin and was sensitive enough to point out small differences between cell lines of the same tissue origin. Therefore, we present this novel application of the aortic ring assay to not only assess the angiogenic properties of candidate cell types for tissue regeneration, but also to identify the most suitable donor-derived cell lines to bank for future use in cell therapy.

For the present study, we selected and compared two human MSC types from different tissue origins. Both cell types were implemented previously to possess proangiogenic properties and their candidacy for regenerative medicinal applications is heavily based on this attribute. The differences observed between commercially available BMSCs (Lonza) expanded in animal serum (FBS)-containing conditions and FTM HUCPVCs expanded in clinically compliant xeno-free media (StemPro) can occur for various reasons. Our aim was to demonstrate that this modified version of the aortic ring assay is robust and sensitive enough to display significant differences in cellular behavior, acknowledging that such differences are inevitably a combination of multiple endogenous and induced characteristics, including the effect of culture conditions. Our laboratory has also investigated the angiogenic properties of FTM HUCPVCs and BMSCs in other, technically less convenient angiogenic assays often involving live animals. Although the results were similar, we aimed to present the possibilities of the approach that we found to offer the most feasible and reliable application we have come across to date. We propose that the aortic ring assay can be developed as a valuable, quantitative prescreening tool for candidate cell lines for regenerative therapy.

## Conclusions

The presented standardized, direct coculture aortic ring assay is capable of elaborating on the differences in angiogenic potential of mesenchymal stem cells in a multiplex, quantitative manner. The functional comparison of young, extraembryonic human MSCs (FTM HUCPVCs) and adult tissue-originated MSCs (BMSCs) clearly demonstrated both the robustness of this novel in vitro analytical approach and the significant merits of using FTM HUCPVCs for in vivo regenerative applications.

## Additional file

Additional file 1: Figure S1. Showing cellular fractions of aortic ring cultures isolated and processed for flow cytometry analysis. Fluorophore-conjugated antibody against human specific cell surface marker (TRA-1-85(APC)) was applied in combination with MSC/pericyte marker (CD146(FITC), **A**) or endothelial marker (CD31(FITC), **B**) specific antibodies. Cell population positive for TRA-1-85 (*y* axis) tested positive for MSC/pericyte marker (CD146 (**A**, Q6)) and slight positivity for endothelial marker (CD31 (**B**, Q1)). This suggests that FTM HUCPVCs maintained their perivascular cell properties in aortic ring cocultures and did not develop an endothelial phenotype. Quadrants defined using isotype controls matching applied primary antibodies. (PDF 91 kb)

## Abbreviations

BMSC: Bone marrow stromal cell
CTG: Cell tracker green
EBM: Endothelial basal media
ECM: Extracellular matrix
EGM: Endothelial growth media
EPC: Endothelial progenitor cell
FGF: Fibroblast growth factor
FTM: First trimester (of gestation)
HUC: Human umbilical cord
HUCPVC: Human umbilical cord perivascular cell
MEM: Minimum essential media
MSC: Mesenchymal stromal cell
NG2: Neural/glial antigen 2
PDGF: Platelet-derived growth factor
PDGFR: PDGF receptor
VCAM-1: Vascular cell adhesion molecule-1
VEGF: Vascular endothelial growth factor
