# Supplementary figures and images for: Angiogenic potency evaluation of cell therapy candidates by a novel application of the in vitro aortic ring assay
Source: Stem Cell Res Ther. 2017 Aug 14;8:184. doi: 10.1186/s13287-017-0631-1 (PMC5557530; doi:10.1186/s13287-017-0631-1)

# Flow cytometry analysis of rat aortic ring, FTM HUCPVC co-culture

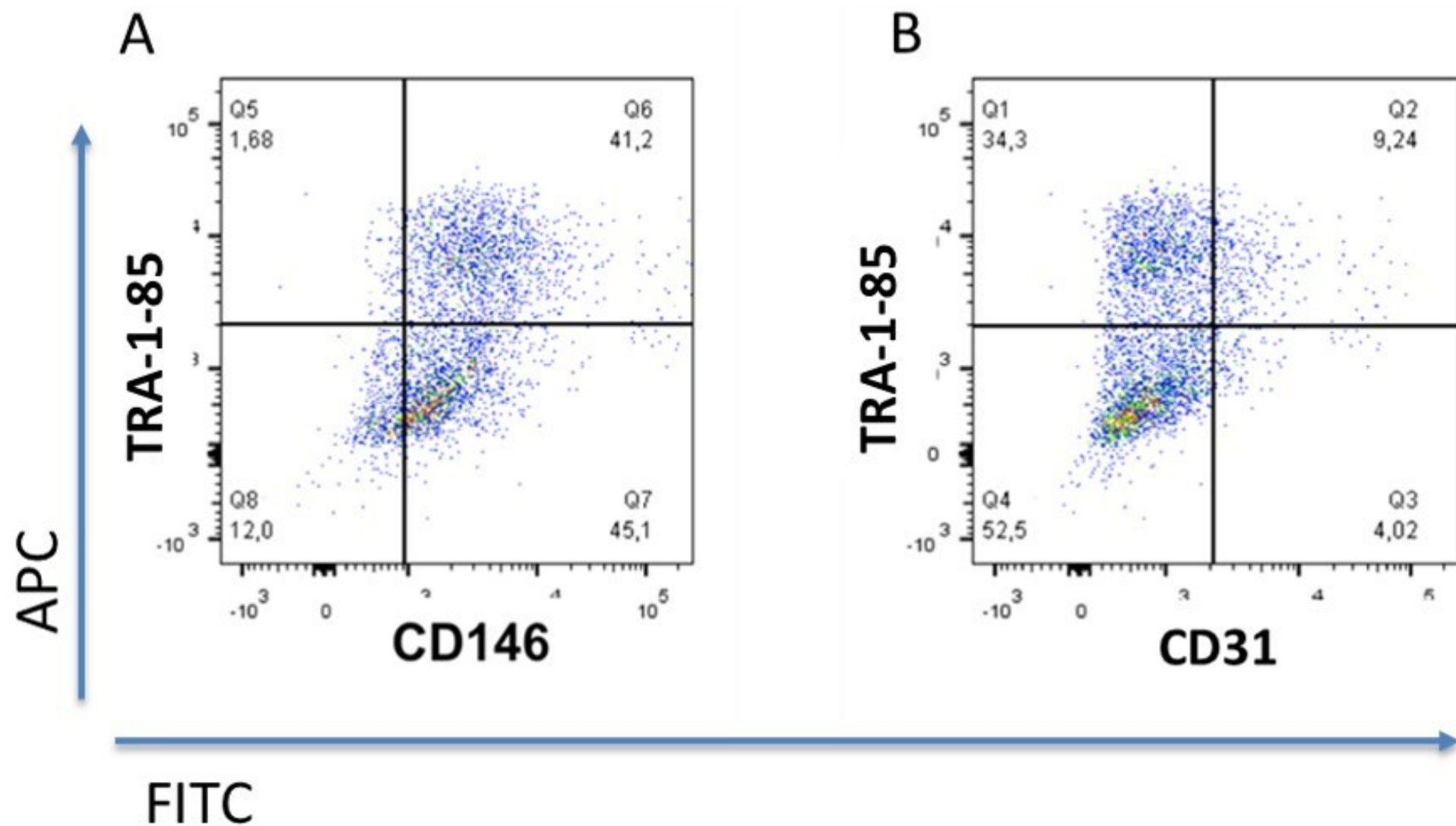

Supplement: Additional file 1: Figure S1. — Showing cellular fractions of aortic ring cultures isolated and processed for flow cytometry analysis. Fluorophore-conjugated antibody against human specific cell surface marker (TRA-1-85(APC)) was applied in combination with MSC/pericyte marker (CD146(FITC), A) or endothelial marker (CD31(FITC), B) specific antibodies. Cell population positive for TRA-1-85 (y axis) tested positive for MSC/pericyte marker (CD146 (A, Q6)) and slight positivity for endothelial marker (CD31 (B, Q1)). This suggests that FTM HUCPVCs maintained their perivascular cell properties in aortic ring cocultures and did not develop an endothelial phenotype. Quadrants defined using isotype controls matching applied primary antibodies. (PDF 91 kb) [file 13287_2017_631_MOESM1_ESM.pdf]
